# Plasma trans-fatty acids levels and mortality: a cohort study based on 1999–2000 National Health and Nutrition Examination Survey (NHANES)

**DOI:** 10.1186/s12944-017-0567-6

**Published:** 2017-09-16

**Authors:** Haibo Li, Qian Zhang, Jiangen Song, Anshi Wang, Yunfei Zou, Lei Ding, Yufeng Wen

**Affiliations:** 1 School of Public Health, Wannan Medical College, No. 22 Western Wenchang Road, Wuhu, 241002 China; 2 Graduate School, Peking Union Medical College, Beijing, 100730 China; 3 Department of Epidemiology, Capital Institute of Pediatrics, Beijing, 100020 China; 4 Department of Epidemiology and Biostatistics, School of Public Health, Anhui Medical University, Hefei, 230032 China

**Keywords:** Trans-fatty acids, Mortality, Cardiovascular diseases, Cancer

## Abstract

**Background:**

Trans-fatty acids (TFAs) occur in small amounts in nature but became widely produced by the food industry. The hazardous effects of different TFA subtypes to human health are controversial. We aimed to evaluate the association of plasma TFAs levels (elaidic acid, vaccenic acid, palmitelaidic acid, and linoelaidic acid) with mortality.

**Methods:**

Utilizing 1999–2000 Nutrition Examination Survey (NHANES) and linked mortality data, we performed a cohort study with 1456 participants and used Cox proportional hazards models and penalized smoothing spline plots to elucidate the relationships between TFAs and all-cause, cardiovascular diseases (CVD) and cancer mortality.

**Results:**

During 16,034 person-years of follow-up, a total of 221 deaths occurred. In the multivariate model, including mutual adjustment for the 4 TFA subtypes, elaidic acid associated with higher all-cause mortality (hazard ratio (HR) = 2.00, 95% confidence interval (CI) = 1.18 to 3.40, fourth quartiles versus second quartiles) and CVD mortality (HR = 1.64, 95% CI = 1.07 to 2.50, per 10 units increase). Higher palmitelaidic acid levels were associated with increased cancer mortality (HR = 2.91, 95% CI = 1.09 to 7.81, fourth quartiles versus second quartiles). A J-shaped pattern was observed in the regression curve of elaidic acid and all-cause mortality, as well palmitelaidic acid and cancer mortality.

**Conclusions:**

Plasma elaidic acid levels are associated with higher risk of all-cause and CVD mortality, and palmitelaidic acid levels are associated with higher cancer mortality in later life. Further studies are needed to investigate current inconsistent results in this field and the possible underlying mechanisms.

**Electronic supplementary material:**

The online version of this article (10.1186/s12944-017-0567-6) contains supplementary material, which is available to authorized users.

## Background

Trans-fatty acids (TFAs), a type of unsaturated fatty acid containing double bonds in the trans-configuration, are not commonly found in nature (ruminant TFAs). However, industrial production of TFAs from vegetable fats since the 1950s was widely increased for use in processed foods, margarine, frying fast foods and commercial baked goods [1]. In industrially-produced TFAs (IP-TFAs), a portion of cis-isomers are converted into trans-isomers during hydrogenation. This conversion stabilizes polyunsaturated oils, protects them against rancidification and keeps them solid at room temperature. TFAs, particularly IP-TFAs, consistently increases the risk for diseases associated with the modern Western lifestyle such as cardiovascular diseases (CVD) [2–4], stroke [5], diabetes [6], Alzheimer’s disease [7], and certain cancers [8]. Therefore, many measures have been adopted to reduce the general population’s intake of TFAs. The Food and Drug Administration announced a final rule that requires food manufacturers to list TFAs on nutrition facts labels in 2003 [9]. As a result of this rule, the US National Health and Nutrition Examination Survey (NHANES) 1999–2000 show that plasma TFA concentrations in American adults declined from 1999 to 2010 [10–12].

An abundance of studies have investigated the adverse health effects of different TFA subtypes and their specific isomers concentrations in blood and have found inconsistent results. For instance, some early studies suggested that high blood levels of TFAs or TFA (all types of isomers, include C18:1 t) intake was associated with increased risk of prostate cancer [13] or coronary heart disease (CHD) [14–16]. However, some studies argued that TFA C18:2 t, but not C18:1 t or C16:1 t, were associated with nonfatal acute myocardial infarction (MI) [17], CHD death [18], total mortality [19] and sudden cardiac arrest [20]. One explanation for these conflicting reports is that most prior studies evaluated only the sum of these TFAs. In addition, it is also debated whether variations of TFAs at low concentrations are associated with adverse outcomes risk. It is unknown how different individual isomers relate to total or cause-specific mortality. Therefore, it is necessary to relate to these different TFA subtypes to key outcomes, such as all-cause, CVD or cancer mortality.

To evaluate the association of plasma TFAs levels with mortality, we conducted a cohort study using data from the NHANES 1999–2000, a large, nationally representative, socioeconomically and ethnically diverse sample of U.S. adults [21]. Our primary goals were to address the following research questions: 1) Are plasma TFAs levels associated with all-cause or cause-specific mortality in adults aged 20–84? 2) What is the highest concentration of TFA that may be harmless? 3) Should the same thresholds be applied to both IP-TFAs and ruminant TFAs? Answering these questions could help comprehensively illuminate the association between TFAs and mortality risk.

## Methods

### Sample and procedures

Each NHANES participant undergoes a household interview and a physical examination in a Mobile Examination Center [21]. More details about NHANES and its methods have been published [22]. The study protocol was approved by the National Center for Health Statistics (NCHS) Ethics Review Board and written informed consent was obtained.

Baseline data for this study were obtained from the publicly released NHANES 1999–2000. It is a cross-sectional sample of about 9965 individuals aged 2 months and older. Of these, 2188 participants were eligible to have their plasma TFAs examined. Given the possibility of survival bias among the extreme elderly, we restricted our analysis to persons who were ≥20 years and <85 years at baseline. Next, 732 were excluded due to missing information on one or more covariates and/or mortality. Finally, 1456 subjects, who met the inclusion criteria, were included in our analysis.

### Mortality

The de-identified and anonymized data of NHANES 1999–2000 participants were linked to longitudinal medicare and mortality data using the NHANES assigned sequence number [23, 24]. Mortality follow-up data are available from the date of survey participation until December 31, 2011. Person-years of follow-up from the interview date ranged from 0.01 to 12.7, with a mean of 11.0. We examined all-cause mortality, as well as mortality due to CVD, cancer, and other diseases including chronic lower respiratory diseases, influenza, pneumonia, liver disease, kidney disease, diabetes, and Alzheimer’s disease. Cause of death was determined using the *International Classification of Diseases, Tenth Revision (ICD-10)*. Cardiovascular death was classified using *ICD-10* codes *I00-I78*, and cancer death was classified using *ICD-10* codes *C00-C97*.

### Trans-fatty acids

Four TFAs (elaidic acid [C18:1 t9], vaccenic acid [C18:1 t11], palmitelaidic acid [C16:1 t9], and linoelaidic acid [C18:2 t9, 12]) were measured in plasma stored at −70 °C, following previously described procedures [25, 26]. Description of laboratory methodology and the quality control protocol is available at https://wwwn.cdc.gov/nchs/data/nhanes/1999-2000/labmethods/TFA_A_trans_fatty_acids_met.pdf.

The four major TFAs examined here are reasonably representative of TFAs in blood [27]. The C18:1 t isomers account for 80%–90% of total TFAs in foods [28], including elaidic acid and vaccenic acid. Elaidic acid (C18:1 9 t) is the major isomer in industrial sources of TFAs. The transisomer of vaccenic acid (C18:1 11 t), a precursor for conjugated linoleic acid, is the major TFA isomer in ruminant fat and is also less frequently found in IP-TFAs [29]. Other TFA isomers include C16:1 t, C18:2 t, C18:3 t, as well as long-chain polyunsaturated TFAs [29] including palmitoleic acid (C16:1 t9) and linolelaidic acid (C18:2 t9, 12). The major sources of palmitoleic acid are ruminant meat and milk, while linolelaidic acid partially comes from hydrogenated oils.

### Other covariates

Demographic data and health history (diabetes, hypertension, coronary heart disease, myocardial infarction, and stroke) were determined by self-report. Race was classified as non-Hispanic white, non-Hispanic black, other Hispanic, Mexican American, or others. Body mass index (BMI), systolic blood pressure (SBP), diastolic blood pressure (DBP), fasting plasma glucose (FPG), triglyceride (TG), total cholesterol (TC), high density lipoprotein (HDL), uric acid (UA) and creatinine (Cr) were measured by standard protocol. Estimation glomerular filtration rate (eGFR) was calculated using the Chronic Kidney Disease Epidemiology Collaboration (CKD-EPI) study equation [30]. Alcohol use was determined by self-reporting (yes or no) whether they have at least 12 servings of any type of alcoholic beverage in any 1 year. Smoking status was categorized as current smoker (frequent or occasional), former smoker, and nonsmoker. Prescription medication use was assessed by self-report and verified by interviewers through examination of medication containers. Diabetes was defined as a fasting glucose value ≥7.0 mmol/L or use of insulin or oral hypoglycemic agent. Hypertension was defined as systolic/diastolic blood pressure ≥ 140/90 mmHg or use of antihypertensive drugs.

### Statistical analysis

We used the R *survey* package [31] to appropriately weight analyses for the complex, multistage sampling design of NHANES. The sample weight, WTMEC2YR (full sample 2-year MEC exam weight) was used to obtain unbiased national estimates. We included cluster and strata variables from demographic datasets in our weighted analyses to account for survey design.

First, we conducted penalized smoothing spline by R package *pspline* [32] to develop hazards ratio (HR) curves to explore the possible nonlinear relationships between TFAs and mortality based on Cox proportional hazards models. In these models, the covariates were adjusted and TFA subtypes were used as continuous variables.

Second, we applied Cox proportional hazards models to explore the relationships between TFA levels and all-cause, CVD and cancer mortality among whole cohorts, with time-at-risk until the first event of interest, censoring at other causes of death in analyses of cause-specific mortality or December 31, 2011. We performed models with baseline TFAs levels as a continuous predictor and as categorical predictor (according to quartiles and used the second quartiles as reference category). In these models, per standard deviation (sd) increase TFAs measure was also to reflect increased risk of mortality. Cox proportional hazards models were first age, and gender adjusted and then adjusted for the covariates listed above. Because different TFA subtypes shared some common dietary sources and were partly intercorrelated [19, 33], associations for one subtype could be confounded by the influence of other subtypes [34]. Therefore, we further conducted analyses with mutual adjustment for the various TFA subtypes.

All *P* values were derived from 2-tailed analyses, with significance accepted at *P* ≤ 0.05. Data management and all analyses were performed using R software program, version 3.1.2 (http://www.R-project.org).

## Results

### Baseline characteristics

At baseline, the mean age ± standard deviation was 49.9 ± 17.9 years. Of the cohort, 701 (48.1%) participants were men and 695 (47.7%) were non-Hispanic white. Baseline TFA concentrations significantly differed by population characteristics (Table 1). In unadjusted cross-sectional analyses, older, overweight or obese, diabetic and non-Hispanic white patients tended to have higher TFA concentrations. Compared with individuals assumed alive, participants assumed deceased had higher elaidic acid and palmitelaidic acid. These two TFAs were associated with lower alcohol consumption. In additoin, elaidic acid and linolelaidic acid associated with smoking status, and participants diagnosed with hypertension had higher linolelaidic acid than those without hypertension (Table 1).

**Table 1:** TFAs concentrations by selected population characteristics of U.S. adults (Age ≥ 20 Years), NHANES 1999–2000

Population characteristics	Number	Unweighted, %	Weighted, %	Elaidic acid (C18:1 t9) Weighted mean (SE) μM	Vaccenic acid (C18:1 t11) Weighted mean (SE) μM	Palmitelaidic acid (C16:1 t9) Weighted mean (SE) μM	Linolelaidic acid (C18:2 t9, 12) Weighted mean (SE) μM
Gender							
Male (ref)	701	48.1	48.8	36.40(1.87)	43.12(1.96)	7.43(0.28)	3.04(0.15)
Female	755	51.9	51.2	37.99(1.41)	41.24(1.36)	7.67(0.15)	3.20(0.11)
Age, y							
20–39 (ref)	509	35	42.8	34.98(1.27)	41.23(1.33)	7.30(0.19)	2.96(0.10)
40–59	429	29.5	36.0	39.26(2.47) ^*^	43.62(2.27)	7.60(0.34)	3.26(0.19) ^*^
60	518	35.5	21.2	38.27(1.36) ^*^	41.57(1.27)	7.97(0.22) ^*^	3.21(0.13) ^*^
Race							
Non-Hispanic White (ref)	695	47.7	75.0	38.97(1.75)	43.83(1.67)	7.97(0.19)	3.31(0.15)
Non-Hispanic Black	222	15.2	8.5	31.52(1.07) ^*^	37.80(1.19) ^*^	6.00(0.29) ^*^	2.39(0.08) ^*^
Mexican American	424	29.1	6.4	37.07(2.31)	41.76(2.39)	7.27(0.31) ^*^	2.94(0.18) ^*^
Other Hispanic	80	5.5	7.1	26.82(2.27) ^*^	33.41(2.26) ^*^	5.87(0.12) ^*^	2.43(0.08) ^*^
Other race / multiracial	35	2.5	3.0	34.49(5.04)	34.28(6.28)	6.05(0.75) ^*^	2.59(0.21) ^*^
BMI, kg/m^2^							
Underweight (<18.5)	24	1.6	2.4	31.68(1.28)	40.01(1.96)	6.88(0.43)	2.28(0.14)
Normal weight (18.5–24.9) (ref)	459	31.5	36.3	33.98(1.60)	39.32(1.62)	7.04(0.20)	2.69(0.11)
Overweight (25–29.9)	490	33.7	31.6	36.21(1.52)	41.59(1.66)	7.36(0.27)	3.21(0.15) ^*^
Obese (>30)	483	33.2	29.7	42.67(1.88) ^*^	46.39(1.79) ^*^	8.43(0.21) ^*^	3.62(0.14) ^*^
Alcohol use							
No (ref)	454	31.2	26.1	39.41(1.22)	43.44(0.89)	8.12(0.16)	3.12(0.12)
Yes	1002	68.8	73.9	36.44(1.67) ^*^	41.71(1.71)	7.35(0.18) ^*^	3.12(0.13)
Smoking status							
Nonsmoker (ref)	720	49.5	46.8	35.58(1.82)	41.08(1.75)	7.53(0.22)	2.94(0.13)
Former	426	29.2	28.9	39.07(1.82) ^*^	43.70(1.71)	8.08(0.26)	3.29(0.16) ^*^
Current	310	21.3	24.3	38.16(1.92)	42.40(2.08)	6.97(0.32)	3.27(0.16) ^*^
Hypertension^a^							
No (ref)	941	64.6	72.4	36.42(1.75)	42.09(1.76)	7.49(0.24)	3.02(0.13)
Yes	515	35.4	27.6	39.3(1.90)	42.35(1.36)	7.71(0.21)	3.38(0.13) ^*^
Diabetes^b^							
No (ref)	1338	91.9	94.9	36.48(1.44)	41.57(1.34)	7.43(0.16)	3.09(0.12)
Yes	118	8.1	5.1	50.89(5.37) ^*^	53.12(3.89) ^*^	9.81(0.71) ^*^	3.79(0.20)^*^
Self-reported cardiovascular diseases history							
No (ref)	1358	93.3	94.7	37.05(1.51)	42.16(1.40)	7.53(0.16)	3.11(0.12)
Yes	98	6.7	5.3	40.16(2.70)	42.03(2.97)	7.92(0.65)	3.31(0.19)
Mortality status, n (%)							
Assumed alive (ref)	1235	84.8	90.0	36.65(1.45)	41.82(1.39)	7.45(0.17)	3.12(0.12)
Assumed deceased	221	15.2	10.0	42.27(3.00) ^*^	45.25(2.86)	8.42(0.44) ^*^	3.18(0.15)
Overall	1456	100	100	37.22(1.44)	42.16(1.33)	7.55(0.15)	3.12(0.11)

^*^ *P* < 0.05 for a significant difference in tran-fatty acids concentrations in the category compared to reference (first category listed) using weighted ANOVA or two-samples t tests

^a^Hypertension was defined as systolic and diastolic blood pressures ≥140/90 mmHg or use of antihypertensive treatment

^b^Diabetes was defined as a fasting glucose value ≥7.0 mmol/L or use of insulin or oral hypoglycemic agent

### Intercorrelations between TFA subtypes

**Fig. 1:**
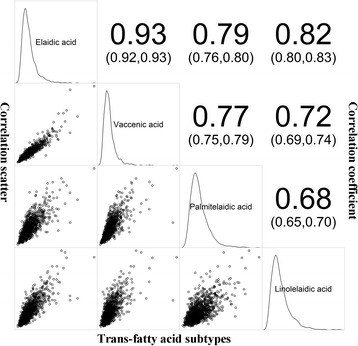
Correlations plot between baseline TFA subtypes. Correlation scatter diagrams in lower left, correlation coefficient (95% confidence interval) in higher right

Distribution of TFAs and intercorrelations between TFA subtypes are presented in Fig. 1. Highly relevant intercorrelations between TFA subtypes were observed and were lowest for elaidic acid and linolelaidic acid (correlation coefficient = 0.68).

### Correlations between TFAs and all-cause mortality

A median follow-up of 11.6 years was obtained from the 1456 subjects. During 16,034 person-years of follow-up, a total of 221 deaths occurred, including 39 and 60 deaths attributed to CVD and cancer, respectively.

**Fig. 2:**
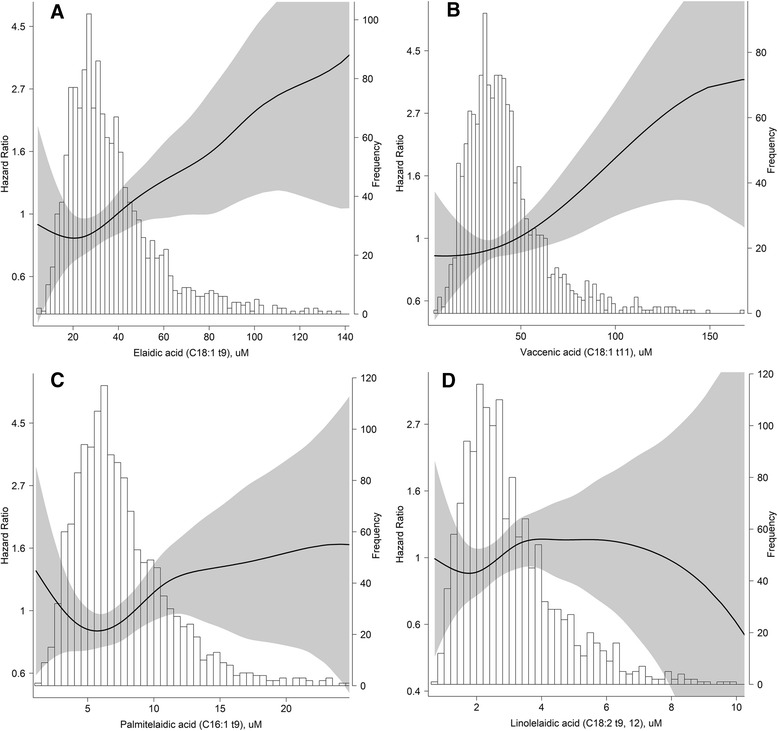
Relationship between baseline TFA subtypes (μM) and incident all-cause mortality and their 95% confidence intervals (shade scope), based on Cox proportional hazards regression adjusted for age, gender, race, body mass index, systolic blood pressure, diastolic blood pressure, fasting plasma glucose, triglyceride, total cholesterol, high density lipoprotein, uric acid, estimation glomerular filtration rate, alcohol use, smoking, and self-reported cardiovascular diseases history at baseline. Histogram of TFA subtypes present in right vertical axis. **a** Elaidic acid (C18:1 t9), **b** Vaccenic acid (C18:1 t11), **c** Palmitelaidic acid (C16:1 t9), **d** Linolelaidic acid (C18:2 t9, 12)

Displayed in Fig. 2, penalized smoothing splines in the multivariate proportional hazards models revealed a nonlinear association between TFA levels and mortality. Also shown in Fig. 2a, the risks of all-cause mortality sharply raised as elaidic acid levels increased after the relatively low level of approximately 25 μmol/L (μM). This risk slightly leveled off, displaying a J-shaped pattern. Figure 2b revealed an approximately linear and positive association between vaccenic acid and all-cause mortality. While a U-shaped curve with a threshold of 6.0 (palmitelaidic acid) μM/2.0 (linolelaidic acid) μM were observed in Fig. 2c, d.

Considering nonlinear correlation patterns between TFA levels and all-cause mortality, we defined the second quartile as the reference category. In age and gender adjusted analysis, elaidic acid was associated with higher all-cause mortality, with 63% higher risk among participants in the top quartile, compared with the second quartile (HR = 1.63, 95%CI = 1.12 to 2.38). Other TFA subtypes were not significantly associated with all-cause mortality upon age and gender adjustment. Further adjusted for potential confounders (race, BMI, SBP, DBP, FPG, TG, TC, HDL, UA, eGFR, alcohol use, smoking, and self-reported CVD history at baseline) did not diminish the effect sizes of elaidic acid (HR = 2.01, 95%CI = 1.30 to 3.08). Also, palmitelaidic acid (HR = 1.82) and linolelaidic acid (HR = 1.49, quartile 3 vs. quartile 2) became significantly associated with all-cause mortality after adjustments. Considering intercorrelations of the TFA subtypes (Fig. 1), we performed further analyses with mutual adjustment to evaluate independent associations. After mutual adjustment, elaidic acid remained associated with all-cause mortality (HR = 2.00, 95%CI = 1.18 to 3.40). Other TFA subtypes were not significantly associated with all-cause mortality with mutual adjustment. In addition, the lowest TFAs quartiles, except for vaccenic acid, were also related to increased mortality. This tendency was fit for all model analysis (Table 2).

**Table 2:** Prospective associations of plasma tran-fatty acids with risk of total mortality (*n* = 1456)

	Events (n/%)	Number at risk (Person-years)	Model 1		Model 2		Model 3	
			Hazard ratio (95% CI)	*P* value	Hazard ratio (95% CI)	*P* value	Hazard ratio (95% CI)	*P* value
Elaidic acid (C18:1 t9), μM								
Per 10 units increase	221 (15.2)	1456 (16034)	1.07(1.02–1.13)	0.010	1.13(1.05–1.22)	0.002	1.30(1.08–1.56)	0.005
Per sd increase			1.17(1.04–1.31)	0.010	1.31(1.11–1.56)	0.002	1.79(1.19–2.67)	0.005
Quartile (Range, μM)								
Quartile 1 (4.5–23.4)	51(14.0)	363(3974)	1.20(0.80–1.78)	0.377	1.35(0.89–2.04)	0.162	1.37(0.89–2.09)	0.154
Quartile 2 (23.5–32.3)	47(12.9)	363(4078)	Reference		Reference		Reference	
Quartile 3 (32.4–44.7)	55(15.0)	366(4048)	1.39(0.93–2.05)	0.104	1.49(1.00–2.22)	0.051	1.48(0.99–2.24)	0.059
Quartile 4 (44.9–238)	68(18.7)	364(3934)	1.63(1.12–2.38)	0.010	2.01(1.30–3.08)	0.002	2.00(1.18–3.40)	0.010
Vaccenic acid (C18:1 t11), μM								
Per 10 units increase	221 (15.2)	1456 (16034)	1.05(0.99–1.09)	0.053	1.08(1.01–1.15)	0.020	0.92(0.80–1.06)	0.241
Per sd increase			1.12(0.99–1.25)	0.053	1.20(1.03–1.40)	0.020	0.81(0.57–1.15)	0.241
Quartile (Range, μM)								
Quartile 1 (4.45–27.9)	54(14.8)	364(3997)	0.93(0.64–1.35)	0.707	0.95(0.64–1.41)	0.811	1.00(0.67–1.49)	0.999
Quartile 2 (27.9–37.5)	55(15.2)	363(4019)	Reference		Reference		Reference	
Quartile 3 (37.6–49.7)	49(13.5)	364(4036)	0.89(0.60–1.31)	0.549	1.04(0.70–1.54)	0.865	0.93(0.62–1.41)	0.743
Quartile 4 (49.8–313)	63(17.3)	365(3982)	1.23(0.85–1.76)	0.271	1.39(0.92–2.12)	0.119	0.99(0.57–1.71)	0.969
Palmitelaidic acid (C16:1 t9), μM								
Per unit increase	221 (15.2)	1456 (16034)	1.02(0.99–1.06)	0.247	1.04(0.99–1.09)	0.106	1.00(0.94–1.07)	0.896
Per sd increase			1.08(0.95–1.23)	0.247	1.16(0.97–1.39)	0.106	1.02(0.80–1.29)	0.896
Quartile (Range, μM)								
Quartile 1 (1.1–5.1)	45(12.4)	363(3989)	1.06(0.71–1.59)	0.758	1.11(0.73–1.69)	0.628	1.15(0.76–1.76)	0.510
Quartile 2 (5.1–6.8)	54(14.8)	365(4060)	Reference		Reference		Reference	
Quartile 3 (6.9–9.4)	55(15.1)	364(4057)	0.95(0.65–1.38)	0.776	1.10(0.74–1.62)	0.639	1.05(0.70–1.57)	0.817
Quartile 4 (9.4–33.1)	67(18.4)	364(3928)	1.35(0.95–1.94)	0.098	1.82(1.19–2.80)	0.006	1.49(0.91–2.43)	0.115
Linolelaidic acid (C18:2 t9, 12), μM								
Per unit increase	221 (15.2)	1456 (16034)	1.04(0.96–1.13)	0.370	1.05(0.92–1.19)	0.487	0.86(0.73–1.02)	0.088
Per sd			1.06(0.93–1.21)	0.370	1.08(0.88–1.32)	0.487	0.79(0.60–1.04)	0.088
Quartile (Range, μM)								
Quartile 1 (0.7–2.0)	55(15.1)	364(3975)	1.16(0.80–1.69)	0.423	1.30(0.88–1.92)	0.187	1.46(0.98–2.19)	0.063
Quartile 2 (2.0–2.7)	56(15.4)	363(4004)	Reference		Reference		Reference	
Quartile 3 (2.7–3.7)	54(14.8)	365(4057)	1.41(0.97–2.06)	0.075	1.49(1.01–2.20)	0.047	1.30(0.87–1.94)	0.200
Quartile 4 (3.7–13.4)	56(15.4)	364(3998)	1.27(0.87–1.85)	0.214	1.26(0.81–1.96)	0.302	0.86(0.52–1.42)	0.561

Model 1: adjusted for age, gender at baseline

Model 2: further adjusted for race, body mass index, systolic blood pressure, diastolic blood pressure, fasting plasma glucose, triglyceride, total cholesterol, high density lipoprotein, uric acid, estimation glomerular filtration rate, alcohol use, smoking, and self-reported cardiovascular diseases history at baseline

Model 3: further adjusted for other trans-fatty acid subtypes

To assess generalizability, we also presented linear relationships between TFA levels and mortality per (10) unit(s)/sd increase. Results showed that elaidic acid positively associated with all-cause mortality in all three models (Per 10 units increase age and gender adjusted HR = 1.07, multivariable adjusted HR = 1.13 in model 2, and equal to 1.30 after mutual adjustment, all *P*-value <0.05). Vaccenic acid was significantly associated with all-cause mortality only observed in model 2 (Per 10 units increase HR = 1.08, 95%CI = 1.01 to 1.15). The other two TFA subtypes were not significant (*P*-value >0.05) (Table 2).

### Correlations between TFAs and CVD and cancer mortality

When we evaluated TFA correlations with CVD and cancer mortality, different nonlinear associations were obtained (Additional file 1: Figure S1 and Additional file 2: Figure S2) for most TFA subtypes. It is worth mentioning that the J-shaped pattern with a threshold of 5.5 μM was more obvious among relationships between palmitelaidic acid and cancer mortality.

In multivariable adjusted analysis, due to fewer outcomes for CVD and cancer mortality, the correlations between TFAs and CVD and cancer mortality became non-significant in most stratification analyses (Tables 3 and 4). The association of elaidic acid with CVD mortality as a continuous variable was statistically significant in model1 and model 3 (Table 3). Palmitelaidic acid was statistically associated with higher cancer mortality, the multivariable adjusted HR was equal to 2.56 among participants in the top quartile, compared with the second quartile, even after mutual adjustment (HR = 2.91, 95%CI = 1.09 to 7.81) (Table 4).

**Table 3:** Prospective associations of plasma tran-fatty acids with risk of cardiovascular diseases mortality (*n* = 1456)

	Events (n/%)	Number at risk (Person-years)	Model 1		Model 2		Model 3	
			Hazard Ratio (95% CI)	*P* value	Hazard Ratio (95% CI)	*P* value	Hazard Ratio (95% CI)	*P* value
Elaidic acid (C18:1 t9), μM								
Per 10 units increase	39(2.7)	1456 (16034)	1.14(1.02–1.27)	0.021	1.12(0.95–1.33)	0.180	1.64(1.07–2.50)	0.023
Per sd increase			1.33(1.04–1.69)	0.021	1.29(0.89–1.88)	0.180	2.96(1.16–7.55)	0.023
Quartile (Range, μM)								
Quartile 1 (4.5–23.4)	6(1.7)	363(3974)	0.66(0.24–1.81)	0.415	1.01(0.34–3.01)	0.983	0.99(0.33–3.04)	0.993
Quartile 2 (23.5–32.3)	10(2.8)	363(4078)	Reference		Reference		Reference	
Quartile 3 (32.4–44.7)	11(3.0)	366(4048)	1.34(0.56–3.18)	0.512	1.31(0.53–3.23)	0.562	1.44(0.56–3.68)	0.447
Quartile 4 (44.9–238)	12(3.3)	364(3934)	1.36(0.58–3.18)	0.473	1.34(0.48–3.71)	0.572	1.67(0.50–5.61)	0.404
Vaccenic acid (C18:1 t11), μM								
Per 10 units increase	39(2.7)	1456 (16034)	1.08(0.98–1.18)	0.135	1.04(0.90–1.20)	0.621	0.75(0.52–1.09)	0.134
Per sd increase			1.20(0.94–1.52)	0.135	1.10(0.76–1.58)	0.621	0.49(0.20–1.24)	0.134
Quartile (Range, μM)								
Quartile 1 (4.45–27.9)	10(2.7)	364(3997)	1.19(0.47–3.00)	0.720	1.35(0.51–3.60)	0.546	1.39(0.51–3.79)	0.516
Quartile 2 (27.9–37.5)	8(2.2)	363(4019)	Reference		Reference		Reference	
Quartile 3 (37.6–49.7)	11(3.0)	364(4036)	1.36(0.55–3.39)	0.509	1.21(0.46–3.18)	0.695	1.22(0.45–3.28)	0.695
Quartile 4 (49.8–313)	10(2.7)	365(3982)	1.35(0.53–3.42)	0.529	1.10(0.37–3.24)	0.864	0.77(0.19–3.11)	0.711
Palmitelaidic acid (C16:1 t9), μM								
Per unit increase	39(2.7)	1456 (16034)	1.05(0.97–1.13)	0.243	1.02(0.92–1.14)	0.707	1.01(0.86–1.18)	0.946
Per sd increase			1.19(0.89–1.58)	0.243	1.08(0.71–1.65)	0.707	1.02(0.55–1.88)	0.946
Quartile (Range, μM)								
Quartile 1 (1.1–5.1)	6(1.7)	363(3989)	0.65(0.24–1.74)	0.388	0.80(0.27–2.31)	0.675	0.84(0.28–2.52)	0.755
Quartile 2 (5.1–6.8)	12(3.3)	365(4060)	Reference		Reference		Reference	
Quartile 3 (6.9–9.4)	8(2.2)	364(4057)	0.61(0.25–1.49)	0.280	0.54(0.21–1.38)	0.202	0.62(0.24–1.63)	0.334
Quartile 4 (9.4–33.1)	13(3.6)	364(3928)	1.17(0.54–2.58)	0.688	1.09(0.39–3.03)	0.866	1.01(0.32–3.20)	0.987
Linolelaidic acid (C18:2 t9, 12), μM								
Per unit increase	39(2.7)	1456 (16034)	1.04(0.85–1.27)	0.720	0.94(0.69–1.28)	0.705	0.74(0.50–1.11)	0.144
Per sd increase			1.06(0.77–1.46)	0.720	0.91(0.55–1.49)	0.705	0.62(0.33–1.17)	0.144
Quartile (Range, μM)								
Quartile 1 (0.7–2.0)	9(2.5)	364(3975)	1.18(0.47–2.99)	0.720	1.08(0.40–2.87)	0.880	1.19(0.44–3.24)	0.732
Quartile 2 (2.0–2.7)	9(2.5)	363(4004)	Reference		Reference		Reference	
Quartile 3 (2.7–3.7)	10(2.7)	365(4057)	1.75(0.70–4.37)	0.230	1.48(0.55–3.95)	0.434	1.35(0.50–3.65)	0.553
Quartile 4 (3.7–13.4)	11(3.0)	364(3998)	1.65(0.68–4.03)	0.271	1.31(0.47–3.65)	0.608	0.89(0.28–2.84)	0.844

Model 1: adjusted for age, gender at baseline

Model 2: further adjusted for race, body mass index, systolic blood pressure, diastolic blood pressure, fasting plasma glucose, triglyceride, total cholesterol, high density lipoprotein, uric acid, estimation glomerular filtration rate, alcohol use, smoking, and self-reported cardiovascular diseases history at baseline

Model 3: further adjusted for other trans-fatty acid subtypes

**Table 4:** Prospective associations of plasma tran-fatty acids with risk of cancer mortality (*n* = 1456)

	Events (n/%)	Number at risk (Person-years)	Model 1		Model 2		Model 3	
			Hazard ratio (95% CI)	*P* value	Hazard ratio (95% CI)	*P* value	Hazard ratio (95% CI)	*P* value
Elaidic acid (C18:1 t9), μM								
Per 10 units increase	60(4.1)	1456 (16034)	1.08(0.98–1.19)	0.137	1.05(0.91–1.23)	0.492	0.88(0.59–1.32)	0.549
Per sd increase			1.18(0.95–1.48)	0.137	1.13(0.80–1.58)	0.492	0.76(0.31–1.86)	0.549
Quartile (Range, μM)								
Quartile 1 (4.5–23.4)	14(3.9)	363(3974)	1.18(0.56–2.52)	0.662	1.42(0.64–3.17)	0.388	1.49(0.66–3.36)	0.336
Quartile 2 (23.5–32.3)	13(3.6)	363(4078)	Reference		Reference		Reference	
Quartile 3 (32.4–44.7)	13(3.6)	366(4048)	1.29(0.60–2.81)	0.514	1.47(0.67–3.24)	0.340	1.42(0.63–3.18)	0.398
Quartile 4 (44.9–238)	20(5.5)	364(3934)	1.86(0.92–3.75)	0.085	1.87(0.82–4.26)	0.139	1.65(0.59–4.63)	0.343
Vaccenic acid (C18:1 t11), μM								
Per 10 units increase	60(4.1)	1456 (16034)	1.07(0.99–1.16)	0.093	1.07(0.94–1.21)	0.326	1.09(0.82–1.46)	0.553
Per sd increase			1.18(0.97–1.44)	0.093	1.17(0.85–1.61)	0.326	1.25(0.60–2.59)	0.553
Quartile (Range, μM)								
Quartile 1 (4.45–27.9)	12(3.3)	364(3997)	0.86(0.39–1.89)	0.708	0.83(0.36–1.91)	0.655	0.82(0.35–1.92)	0.650
Quartile 2 (27.9–37.5)	13(3.6)	363(4019)	Reference		Reference		Reference	
Quartile 3 (37.6–49.7)	15(4.1)	364(4036)	1.18(0.56–2.48)	0.667	1.41(0.66–3.03)	0.374	1.46(0.66–3.25)	0.351
Quartile 4 (49.8–313)	20(5.5)	365(3982)	1.65(0.82–3.31)	0.162	1.73(0.77–3.88)	0.184	2.03(0.68–6.03)	0.205
Palmitelaidic acid (C16:1 t9), μM								
Per unit increase	60(4.1)	1456 (16034)	1.05(1.00–1.12)	0.064	1.06(0.97–1.15)	0.176	1.06(0.95–1.18)	0.314
Per sd increase			1.23(0.99–1.52)	0.064	1.25(0.91–1.71)	0.176	1.24(0.81–1.90)	0.314
Quartile (Range, μM)								
Quartile 1 (1.1–5.1)	13(3.6)	363(3989)	1.34(0.61–2.96)	0.467	1.55(0.67–3.57)	0.302	1.56(0.68–3.61)	0.295
Quartile 2 (5.1–6.8)	12(3.3)	365(4060)	Reference		Reference		Reference	
Quartile 3 (6.9–9.4)	13(3.6)	364(4057)	1.00(0.46–2.20)	0.991	1.30(0.57–2.98)	0.532	1.34(0.57–3.11)	0.500
Quartile 4 (9.4–33.1)	22(6.0)	364(3928)	1.97(0.97–3.98)	0.059	2.56(1.09–6.00)	0.031	2.91(1.09–7.81)	0.034
Linolelaidic acid (C18:2 t9, 12), μM								
Per unit increase	60(4.1)	1456 (16034)	1.12(0.97–1.30)	0.128	1.07(0.85–1.35)	0.562	1.05(0.75–1.47)	0.762
Per sd increase			1.20(0.95–1.51)	0.128	1.11(0.77–1.61)	0.562	1.09(0.63–1.86)	0.762
Quartile (Range, μM)								
Quartile 1 (0.7–2.0)	14(3.8)	364(3975)	1.16(0.55–2.44)	0.694	1.50(0.68–3.28)	0.311	1.58(0.71–3.53)	0.263
Quartile 2 (2.0–2.7)	14(3.9)	363(4004)	Reference		Reference		Reference	
Quartile 3 (2.7–3.7)	16(4.4)	365(4057)	1.85(0.89–3.83)	0.098	2.00(0.93–4.30)	0.077	1.97(0.90–4.33)	0.089
Quartile 4 (3.7–13.4)	16(4.4)	364(3998)	1.61(0.78–3.33)	0.199	1.33(0.57–3.12)	0.514	1.24(0.46–3.34)	0.672

Model 1: adjusted for age, gender at baseline

Model 2: further adjusted for race, body mass index, systolic blood pressure, diastolic blood pressure, fasting plasma glucose, triglyceride, total cholesterol, high density lipoprotein, uric acid, estimation glomerular filtration rate, alcohol use, smoking, and self-reported cardiovascular diseases history at baseline

Model 3: further adjusted for other trans-fatty acid subtypes

## Discussion

We analyzed the association between plasma TFA levels and mortality in the U.S. population. After an average 11.0-year follow-up, we obtained three main findings. 1) Elaidic acid was most strongly associated with higher risk of all-cause or CVD-related mortality, even after adjustment for potential confounders or other TFA subtypes. Regression between elaidic acid and all-cause mortality revealed a J-shaped curve with a threshold elaidic acid level of 25 μM. 2) Vaccenic acid, palmitelaidic acid and linolelaidic acid levels were all significantly associated with all-cause mortality only after multivariate adjustment, but not after mutual adjustment for other TFAs. 3) The J-shaped pattern with a threshold of 5.5 μM is most obvious in the relationship between palmitelaidic acid levels and cancer mortality.

### TFAs and mortality

Several studies report conflicting results regarding associations between plasma TFAs levels and total or CVD mortality [18, 19, 35, 36]. Recently, a sponsored, community-based and multicenter prospective cohort study of older American adults, called Cardiovascular Health Study (CHS) [19], assessed whether plasma phospholipid compositions of TFA subtypes and their isomers are associated with total, cardiovascular and nonvascular mortality. The 31,494 person-years of follow-up from CHS shows the C18:2 t isomer is significantly associated with increased risk of total mortality (extreme quintile HR = 1.23). This association may principally be due to its strong positive association with CVD-related death (HR = 1.40). Interestingly, neither C16:1 t nor C18:1 t isomers were significantly associated with study outcomes. Additionally, no significant or inverse associations between blood TFA levels (total, C16:1 t or C18:1 t) and CVD or all-cause mortality has been observed in other cohort studies [35, 36].

In contrast to previous cohort findings, our study shows elaidic acid (C18:1 9 t) is most strongly associated with higher risks of all-cause and CVD-related mortality. Our findings are biologically plausible. TFA C18:1 t is the major contributor to total TFA consumption from partially hydrogenated vegetable oil, which has been consistently linked to adverse lipid effects in trials [2, 37]. Also, TFAs can increase markers of inflammation [38, 39], promote thrombogenesis and impair flow-mediated dilatation [40]. More recently, human endothelial cells treated with TFAs were found to decrease the production of the vasodilator nitric oxide [41]. Self-reported dietary questionnaires show TFA C18:1 t can elevate the CHD risk [14–16]. Nevertheless, prior biomarker studies [17–19, 34, 42] show no significant or inverse associations of plasma C18:1 t levels with sudden cardiac arrest, CHD or mortality. These contradictory findings may be partially due to the health effects of C18:1 t evaluated in sum in most previous studies that probably mask the effects. It must be noted, the high intercorrelation of C18:1 t isomers preclude their separate evaluation.

Although several studies [17–19, 34, 42, 43] show total C18:2 t relates to higher risk of adverse outcomes, the associations of vaccenic acid, palmitelaidic acid and linolelaidic acid with all-cause mortality found in the present study may be masked by other TFAs or may be a chance finding. Together with previous research, we cautiously conclude that IP-TFAs compared to ruminant TFAs may have greater health hazards in all-cause and CVD-related mortality.

Our study observed a relationship between palmitelaidic acid and cancer mortality. This finding may be supported by the results from North Carolina Colon Cancer Study II [44], which suggests high-TFA consumption is positively associated with distal colorectal cancer among white people. Relative to the lowest quartile, the adjusted odds ratio of the fourth quartile of C16:1 t consumption is 1.57 (95%CI: 1.14 to 2.17). Potential mechanisms may be TFAs could promote colonic mucosa irritation [45], insulin sensitivity [46] and cell proliferation [47, 48], which are positively associated with the incidence of cancers. Nevertheless, further research is needed to uncover why palmitelaidic acid, rather than other TFAs, is associated with cancer mortality.

Higher mortality rates are not restricted to people within the third or fourth quartile of TFA levels, but are also extended to those with lower TFA concentrations. J-shaped or U-shaped associations between plasma TFA levels and all-cause mortality were observed in all tested TFAs, except vaccenic acid. For instance, subjects within the first quartile of elaidic acid levels have 1.35-fold higher risk of all-cause mortality compared to those with median levels. One possible explanation is that low TFA levels are linked to malnutrition, economic poverty or inadequate medical services, which may increase the risk of death. These J- and U-shaped associations should be investigated in detail.

### Strengths and limitations

This study’s strengths lie in its prospective design and a reasonable follow-up duration (11.0 years on average) in the non-institutionalized U.S. population. Second, the measurements of TFA exposure, mortality and other covariates are generally reliable, thanks to the guidance of the U.S. National Center for Health Statistics. Third, measurements of blood TFA levels are generally more reliable and comprehensive than self-reported intake assessment, which are limited in the ability to accurately reflect the nutrient consumption of individuals due to measurement error, recall bias, selective report, and incompleteness of food composition databases [49]. Fourth, we separately evaluated 4 major TFAs and their specific isomers, and thereby provided an accurate and reasonable proof of relationship between specific TFAs and mortality. Furthermore, a penalized smoothing spline model was conducted to develop hazard ratio curves and thereby explore the possible nonlinear relationships between TFAs and mortality. In addition, our ability to adjust confounders increased by prospectively collecting primary covariates including blood lipid parameters using standard methods.

This study has some potential limitations that deserve consideration. First, the measurements of plasma TFA levels were cross-sectional at baseline and may not accurately reflect the long-term blood TFA status. Second, we were unable to illustrate the influences of TFA status on the mortality caused by specific diseases (e.g. Alzheimer’s disease and bowel cancer) due to insufficient data. Finally, given a low event rate, the statistical power in some analyses were low.

## Conclusion

In the current study, we did observe plasma elaidic acid levels are associated with higher risk of all-cause and CVD mortality, and palmitelaidic acid levels are associated with higher cancer mortality in later life. TFA levels are nonlinearly associated with mortality. These findings highlight scientific knowledge that different TFA subtypes may influence total and cause-specific mortality. Further studies are needed to investigate current inconsistent findings in this field and the underlying mechanisms.

## Additional files

Additional file 1: Figure S1. Relationship between baseline TFA subtypes (μM) and incident cardiovascular diseases mortality and their 95% confidence intervals (shade scope), based on Cox proportional hazards regression adjusted for age, gender, race, body mass index, systolic blood pressure, diastolic blood pressure, fasting plasma glucose, triglyceride, total cholesterol, high density lipoprotein, uric acid, estimation glomerular filtration rate, alcohol use, smoking, and self-reported cardiovascular diseases history at baseline. Histogram of TFA subtypes present in right vertical axis. (A) Elaidic acid (C18:1 t9), (B) Vaccenic acid (C18:1 t11), (C) Palmitelaidic acid (C16:1 t9), (D) Linolelaidic acid (C18:2 t9, 12). (TIFF 1842 kb)

Additional file 2: Figure S2. Relationship between baseline TFA subtypes (μM) and incident cancer mortality and their 95% confidence intervals (shade scope), based on Cox proportional hazards regression adjusted for age, gender, race, body mass index, systolic blood pressure, diastolic blood pressure, fasting plasma glucose, triglyceride, total cholesterol, high density lipoprotein, uric acid, estimation glomerular filtration rate, alcohol use, smoking, and self-reported cardiovascular diseases history at baseline. Histogram of TFA subtypes present in right vertical axis. (A) Elaidic acid (C18:1 t9), (B) Vaccenic acid (C18:1 t11), (C) Palmitelaidic acid (C16:1 t9), (D) Linolelaidic acid (C18:2 t9, 12). (TIFF 1820 kb)

## Abbreviations

BMI: Body mass index
CHD: Coronary heart disease
CI: Confidence interval
Cr: Creatinine
CVD: Cardiovascular diseases
DBP: Diastolic blood pressure
eGFR: Estimation glomerular filtration rate
FPG: Fasting plasma glucose
HDL: High density lipoprotein
HR: Hazard ratio
ICD: International Classification of Diseases
IP-TFAs: Industrially produced TFAs
MI: Myocardial infarction
NHANES: Nutrition Examination Survey
SBP: Systolic blood pressure
sd: Standard deviation
TC: Total cholesterol
TFAs: Tran-fatty acids
TG: Triglyceride
UA: Uric acid
μM: μmol/L
