# Supplementary figures and images for: Plasma trans-fatty acids levels and mortality: a cohort study based on 1999–2000 National Health and Nutrition Examination Survey (NHANES)
Source: Lipids Health Dis. 2017 Sep 16;16:176. doi: 10.1186/s12944-017-0567-6 (PMC5603045; doi:10.1186/s12944-017-0567-6)

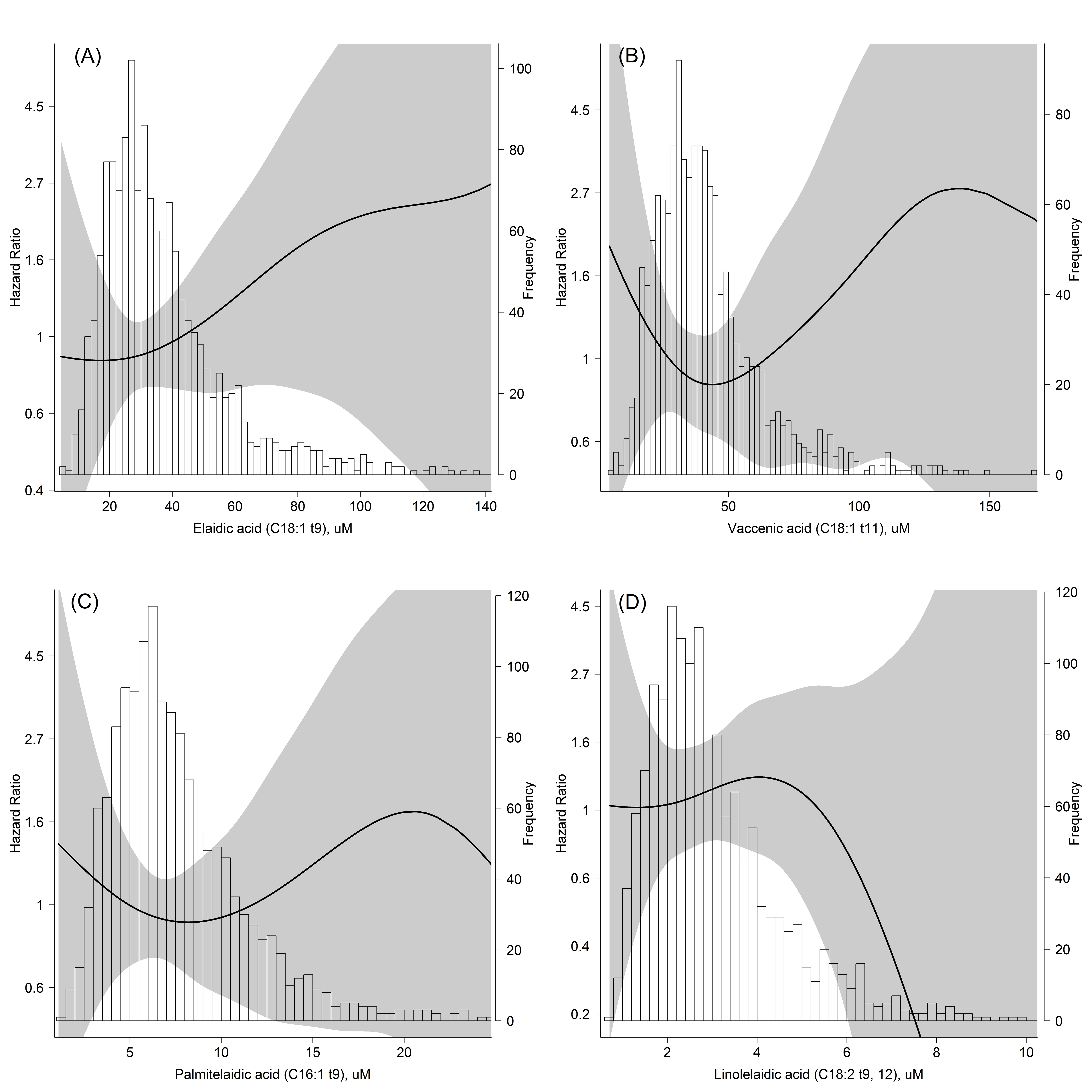

Supplement: Supplementary file 1 — Relationship between baseline TFA subtypes (μM) and incident cardiovascular diseases mortality and their 95% confidence intervals (shade scope), based on Cox proportional hazards regression adjusted for age, gender, race, body mass index, systolic blood pressure, diastolic blood pressure, fasting plasma glucose, triglyceride, total cholesterol, high density lipoprotein, uric acid, estimation glomerular filtration rate, alcohol use, smoking, and self-reported cardiovascular diseases history at baseline. Histogram of TFA subtypes present in right vertical axis. (A) Elaidic acid (C18:1 t9), (B) Vaccenic acid (C18:1 t11), (C) Palmitelaidic acid (C16:1 t9), (D) Linolelaidic acid (C18:2 t9, 12). (TIFF 1842 kb) [file 12944_2017_567_MOESM1_ESM.tif]

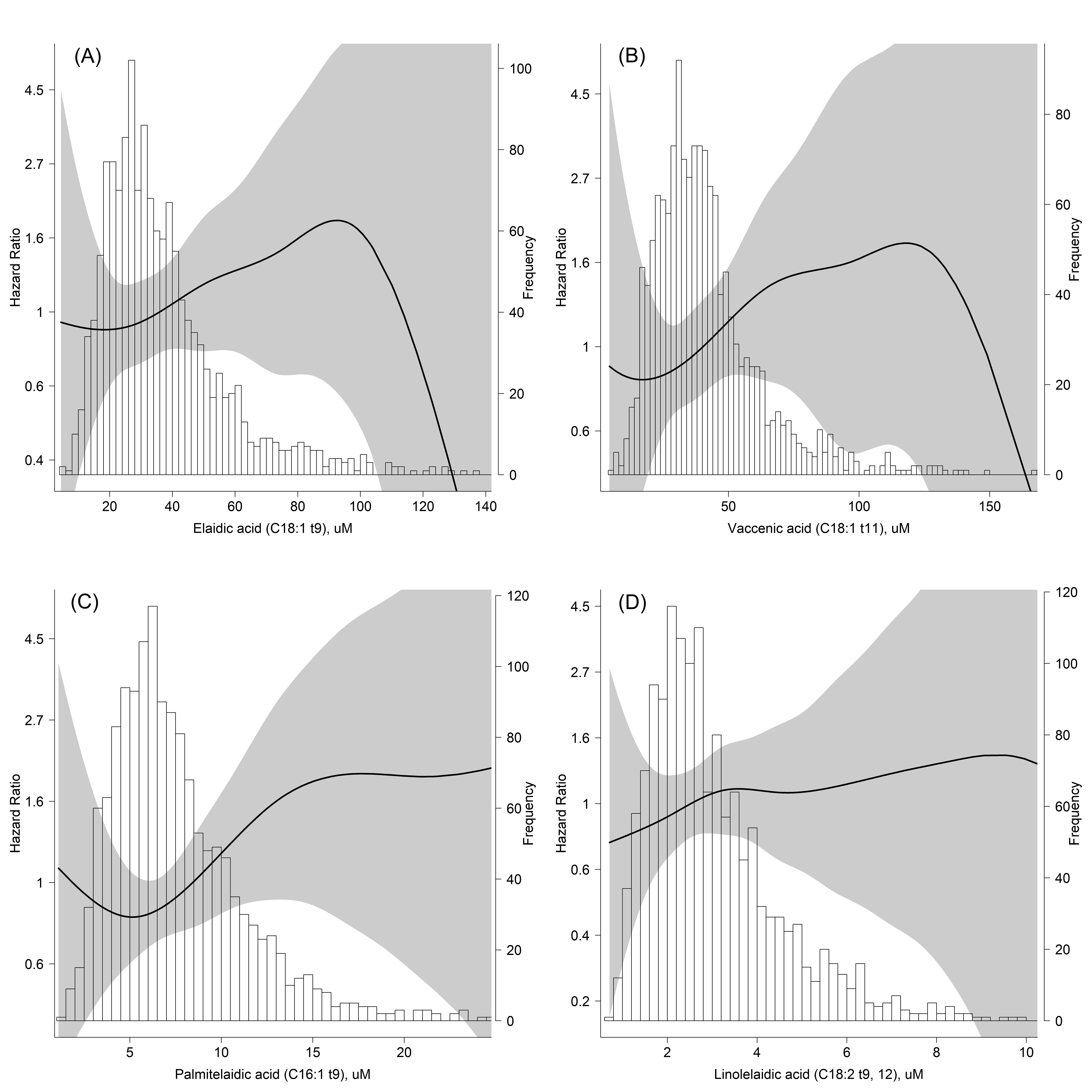

Supplement: Supplementary file 2 — Relationship between baseline TFA subtypes (μM) and incident cancer mortality and their 95% confidence intervals (shade scope), based on Cox proportional hazards regression adjusted for age, gender, race, body mass index, systolic blood pressure, diastolic blood pressure, fasting plasma glucose, triglyceride, total cholesterol, high density lipoprotein, uric acid, estimation glomerular filtration rate, alcohol use, smoking, and self-reported cardiovascular diseases history at baseline. Histogram of TFA subtypes present in right vertical axis. (A) Elaidic acid (C18:1 t9), (B) Vaccenic acid (C18:1 t11), (C) Palmitelaidic acid (C16:1 t9), (D) Linolelaidic acid (C18:2 t9, 12). (TIFF 1820 kb) [file 12944_2017_567_MOESM2_ESM.tif]
